# Blackleg (*Leptosphaeria maculans*) Severity and Yield Loss in Canola in Alberta, Canada

**DOI:** 10.3390/plants5030031

**Published:** 2016-07-20

**Authors:** Sheau-Fang Hwang, Stephen E. Strelkov, Gary Peng, Hafiz Ahmed, Qixing Zhou, George Turnbull

**Affiliations:** 1 Crop Diversification Centre North, Alberta Agriculture and Forestry, Edmonton, AB T5Y 6H3, Canada; hafiz.ahmed@gov.ab.ca (H.A.); qixing.zhou@gov.ab.ca (Q.Z.); george.turnbull@gov.ab.ca (G.T.); 2 Department of Agricultural, Food and Nutritional Science, University of Alberta, Edmonton, AB T6G 2P5, Canada; 3 Saskatoon Research and Development Centre, Agriculture and Agri-Food Canada, Saskatoon, SK S7N 0X2, Canada; gary.peng@agr.gc.ca

**Keywords:** canola, *Leptosphaeria maculans*, blackleg, yield loss model

## Abstract

Blackleg, caused by *Leptosphaeria maculans*, is an important disease of oilseed rape (*Brassica napus* L.) in Canada and throughout the world. Severe epidemics of blackleg can result in significant yield losses. Understanding disease-yield relationships is a prerequisite for measuring the agronomic efficacy and economic benefits of control methods. Field experiments were conducted in 2013, 2014, and 2015 to determine the relationship between blackleg disease severity and yield in a susceptible cultivar and in moderately resistant to resistant canola hybrids. Disease severity was lower, and seed yield was 120%–128% greater, in the moderately resistant to resistant hybrids compared with the susceptible cultivar. Regression analysis showed that pod number and seed yield declined linearly as blackleg severity increased. Seed yield per plant decreased by 1.8 g for each unit increase in disease severity, corresponding to a decline in yield of 17.2% for each unit increase in disease severity. Pyraclostrobin fungicide reduced disease severity in all site-years and increased yield. These results show that the reduction of blackleg in canola crops substantially improves yields.

## 1. Introduction

Blackleg (phoma stem canker) caused by *Leptosphaeria maculans* (Desm.) Ces. & de Not. (anamorph *Phoma lingam* (Tode ex. Fr.) Desm.) is an important disease of canola (oilseed rape, rapeseed; *Brassica napus* L., *B. juncea* (L.) Czern., *B. rapa* L.) worldwide [1,2,3,4]. The disease has been reported to be a major constraint to the production of canola/oilseed rape in Australia, Canada, and Europe [1], and severe epidemics have resulted in heavy yield losses [5,6]. The severity of blackleg epidemics and the extent of yield losses in canola vary greatly, depending on variation in weather, geographic region, and the cultivars grown. An estimated average yield loss of <10% with maximum losses of 30%–50% have been documented as a result of blackleg development in oilseed rape crops [7,8,9]. In France, yield losses of 5%–20% were reported in winter oilseed rape [10], while losses of 8%–29% were documented in the UK from 1987 to 1995 [2].

**Figure 1:**
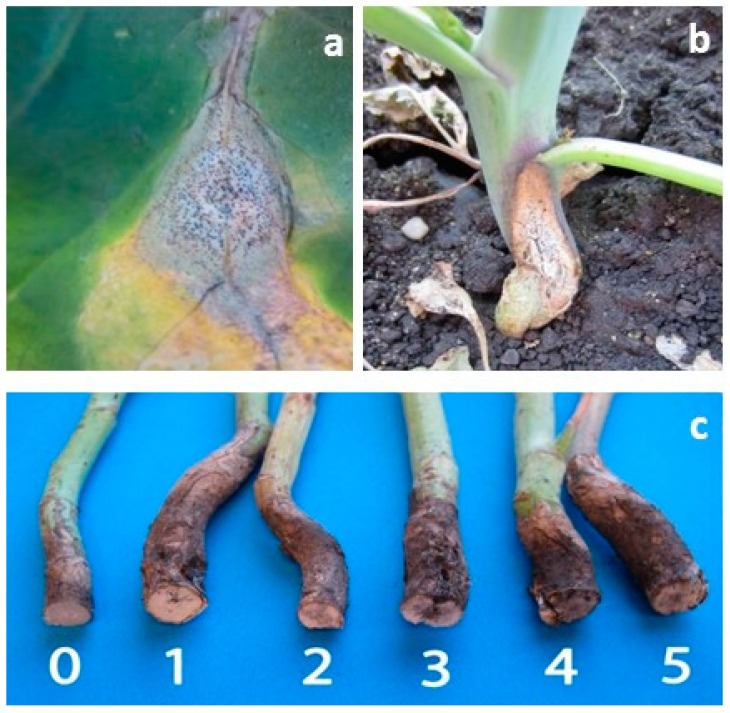
Blackleg lesion on a canola leaf showing pycnidia (**a**); blackleg canker on the lower stem of a mature plant (**b**); rating scale used to assess blackleg disease severity on canola, showing stem cross-sections (**c**).

*Leptosphaeria maculans* survives as a saprophyte on canola stubble [11,12]. The fungus reproduces sexually and releases ascospores in the spring, which serve as the primary inoculum [1]. Blackleg infections may occur at any stage of crop development. On the leaves, the greyish lesions that develop as a result of infection are round to irregularly shaped, and are usually covered with numerous small, black pycnidia (Figure 1). The pycnidia release pycnidiospores for secondary spread of the disease. Hypocotyl infection produces a constriction in the stem above the ground and below the first leaves. The blackleg lesions on hypocotyls can cause severe seedling blight and reduce stand establishment [8]. On older plants, infections usually occur at the base of the stem, or at points of leaf attachment (Figure 1). The stem becomes girdled, and plants ripen prematurely. Severe stem canker results in lodging of the plants and serious yield losses [9,13,14].

Blackleg disease severity can be influenced by manipulating farming practices. Studies have shown that early sowing of the crop reduced the incidence of crown cankers, while high nitrogen availability during the early crop growth stages increased crown canker severity in winter oilseed rape in France [10]. Other cultural practices, such as residue management (e.g., burying infected debris after crop harvest) and maintaining sufficiently long crop rotations (a 4-year interval between oilseed rape crops is usually recommended) will reduce the risk of exposure to primary inoculum produced from infected stubble [1,15,16]. Epidemics of blackleg are generally initiated by airborne ascospores [11,12], which can disperse to a distance of 25 m from the inoculum source [17]. Based on these data, it was recommended that canola crops should be planted at least 50 to 100 m from each other to restrict transmission of *L. maculans* [17]. Australian canola crop management guides recommend a separation of 500 m between canola crops and inoculum sources [18]. Blackleg also can be managed by chemical and biological control methods. Fungicidal treatment of seeds, soil, or foliage is used to control blackleg in many canola growing regions of the world [1]. In western Canada, foliar application of fungicides provides only a limited benefit to canola yield [1,19]. More research is needed on fungicides with new modes of action and the timing of their applications, in order to improve the effectiveness of foliar fungicide applications in the control of diseases such as blackleg.

In Canada, blackleg has been managed largely by the deployment of resistant cultivars [1,20]. However, in recent years the virulence of *L. maculans* populations has shifted [21,22], resulting in the erosion of genetic resistance. This loss of resistance became evident in 2012, when many canola crops sown to resistant and moderately resistant cultivars were heavily infected with blackleg and suffered severe yield losses. In addition, restrictions on canola seed shipments to China caused by seedborne blackleg also have resulted in economic losses [23]. A proactive approach is needed to prevent a more widespread re-emergence of blackleg. While cultural practices such as less intensive rotations are recommended to reduce disease pressure and prolong the effectiveness of genetic resistance [20,22], economic incentives often lead farmers to grow higher value crops such as canola in short rotations. Understanding disease-yield relationships is a prerequisite for assessing the agronomic efficacy and economic benefits of control methods [24]. Therefore, the establishment of a clear relationship between the severity of blackleg infection and the extent of yield loss will assist producers in making informed crop management decisions, by helping them to estimate the possible economic impact of different levels of disease. The objective of this study was to determine the relationship between blackleg disease severity and the yield of canola under conditions in western Canada.

## 2. Results

### 2.1. Cultivar Resistance and Blackleg of Canola

An analysis of variance showed a significant effect of cultivar on the number of pods and yield as well as on blackleg severity. As expected, the disease severity in the resistant canola hybrid “46S53RR” and in the moderately resistant hybrid “1950RR” was lower than in the susceptible cultivar “Westar” over the three site-years (Table 1). The number of pods per plant was greatest for “46S53RR”, intermediate for “1950RR”, and lowest for “Westar”, and these differences were significant. Similarly, the yields were significantly greater for “46S53RR” and “1950RR” and lowest for “Westar” (Table 2).

In an additional trial, analysis of variance revealed a significant effect of cultivar on blackleg severity, pod number, and seed yield. The blackleg severity in the moderately resistant hybrid “Invigor 5440” was lower (Table 1), and pod number was 81% higher, than in “Westar” (Table 2). The seed yield in “Invigor 5440” also was 1.05 t·ha^−1^ greater than in “Westar”.

**Figure 2:**
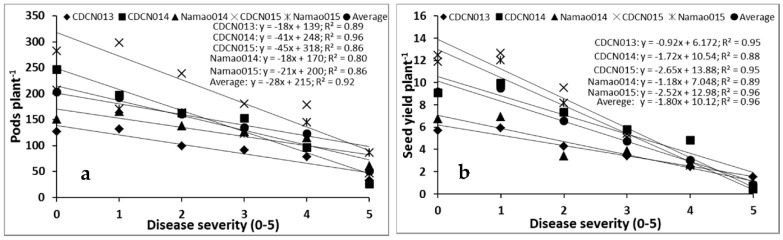
Relationship between blackleg and pods per plant (**a**) and blackleg and seed yield per plant (**b**) in the cultivar “Westar” under field conditions. Data were collected over five site years at two locations in Alberta (Edmonton, 2013–2015 and Namao, 2014 and 2015). Each point represents the mean of four replications × five site-years. Blackleg severity was assessed on a 0–5 scale, where: 0 = no disease and 5 = death of the plant (see text).

Regression analysis showed that pod number and seed yield declined as disease severity increased (Figure 2) in all site-years. The regression model for pods per plant versus disease severity was: y = −28x + 215 (*R^2^* = 0.92) (Figure 2a), and for seed yield per plant versus disease severity, the regression model was: y = −1.80x + 10.12 (*R^2^* = 0.94) when averaged over all site-years (Figure 2b) for the cultivar “Westar”. There were considerable variations in the coefficients of the equations among the site-years, with pod number decreasing by 18–45 per plant and seed yield decreasing by 0.92–2.65 g per plant for every unit increase in disease severity (Figure 2a,b).

**Figure 3:**
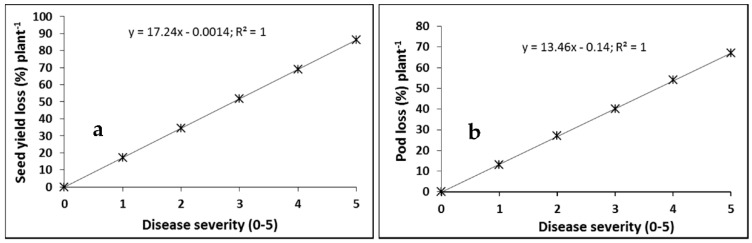
Relationship between blackleg severity and yield loss in the cultivar “Westar” under field conditions at Edmonton, and Namao, Alberta, in 2013–2015. Each point represents the mean of four replications × five site-years. The yield loss data were derived using the y-intercept in the equation averaged over 5 site-years in Figure 2b, which was taken as an estimation of the yield of the cultivar “Westar” in the absence of disease. The data points were transformed into the percentage of that yield (% maximum yield).

In addition, regression of the data transformed into percent yield losses resulted in estimated yield losses in “Westar” of 17.2% (Figure 3a) and pod losses of 13.0% (Figure 3b) for each 1 unit increase in blackleg severity, when averaged over five site-years (2013–2015).

### 2.2. Foliar Fungicide Application and Blackleg

**Figure 4:**
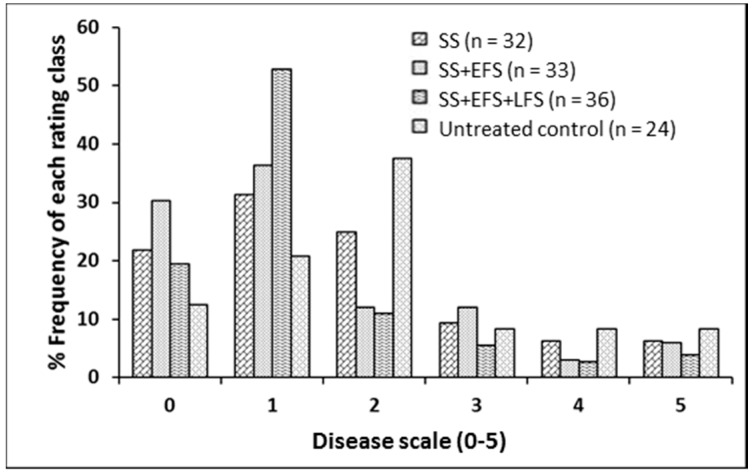
Frequency of plants of the canola hybrid “1950RR” rated 0–5 on a blackleg disease severity scale under different fungicide treatment regimes. The treatments consisted of Headline (pyraclostrobin) application at: seedling stage (SS), SS + early flowering stage (EFS), SS + EFS + late flowering stage (LFS), and an untreated control. Plants were rated on a 0–5 scale, where: 0 = no disease in a cross-section of the stem base; 1 = decay on <25% of the cross-sectional area of the crown; 2 = decay on 25%–50% of the cross-section; 3 = decay on 51%–75% of the cross-section; 4 = decay on >75% of the cross section; and 5 = death of the plant [17]. The experiments were conducted at Edmonton (Crop Diversification Centre North; CDCN) in 2014 and 2015, and at Namao, Alberta, in 2014.

Analysis of variance showed a significant fungicide spray effect on plant count, pods, yield, and disease severity. Fungicide spray schedules improved plant count, pods per plant, and seed yield compared with the unsprayed control (Table 3). Blackleg severity was reduced, relative to the unsprayed control, when the fungicide (Headline, pyraclostrobin, BASF Canada, Mississauga, ON) treatment was applied at the seedling stage + early flowering stage + late flowering stage (Table 4). The frequency of plants rated 0–5 on the blackleg disease severity scale under each fungicide treatment regime is shown in Figure 4.

## 3. Discussion

This study showed that the yield of resistant and moderately resistant canola hybrids was greater relative to that of a susceptible canola cultivar when plots were inoculated with *L. maculans*. This yield difference may not be a function only of disease severity; the inherent yield potential differences among the varieties may have contributed to the variation in the seed yield, since hybrids are typically designed to produce higher yields (due to heterosis) than open-pollenated cultivars. However, since “Westar” is an obsolete cultivar, no direct yield comparisons are available. The cropping of resistant cultivars is the most effective and economic disease management strategy, but the virulence of *L. maculans* populations has shifted in western Canada [21,22]. As a consequence, severe blackleg and yield losses have been observed on some cultivars that were previously considered to be resistant or moderately resistant.

Yield decreased linearly with increasing disease severity. Hall [11] reviewed several studies that described the relationship between blackleg disease severity and yield loss in oilseed rape, and found that most studies showed a linear relationship between seed yield and disease severity. For example, McGee et al. [25] sorted plants into two classes: one that included plants that were severely diseased, while the other included healthy and moderately diseased plants collected from commercial fields. They showed that crop loss was a function of incidence of severely diseased plants, and the relationship was expressed by the equation: L = 0.7D − 2.0 (r = 0.97), where L = % crop yield loss and D = % incidence of severely diseased plants. In the present study, the data were obtained from small plot experiments, precluding collection of a sample sufficiently large to fit the model of McGee et al. [25]. In another study, Rempel et al. [26] scored the severity of internal necrosis of crowns on a 0–4 scale and showed a negative linear relationship between yield and disease severity. They reported that seed yield was reduced by 18% for each unit increase in disease severity. Our study found that seed yield per plant was reduced by 17.2% (Figure 3a) for each unit increase in disease severity, which is in agreement with Rempel et al. [26]. In another study, Peters [27] reported that yield decreased as the severity of crown necrosis increased, and Assabgui et al. [28] expressed this relationship by means of logarithmic equations. In the UK, a yield loss model for stem canker and light leaf spot (*Pyrenopeziza brassicae* Sutton and Rawl) was estimated in relation to disease incidence on stems before harvest [29,30] and the area under the disease progress curve (AUDPC) [31] at early growth stages, respectively. In the current study, similar to [26], single point disease severity at maturity of the crop was used to estimate the yield loss.

The current study showed that a negative linear relationship exists between pod number and disease severity, as well as between seed yield and disease severity. However, the plants with no disease (zero disease severity) had poor growth relative to the plants with a disease score of one, and therefore had lower pod numbers and meagre seed yield. The lower pod numbers and the seed yield associated with plants with a zero disease severity rating may have reflected the late emergence of the plants that escaped *L. maculans* infection. Although the models showed that the relationship between disease severity and seed yield is linear and negative, the total yield loss under field conditions would be dependent on the proportion of plants in each disease category. The higher the proportion of plants with greater disease severity, the greater the expected yield loss. The proportion of plants with a disease severity of 4 to 5 in the present fungicide study ranged from 7% to 16% over all of the trials. Using the yield loss model (L = 0.7D − 2.0) of McGee et al. [25], the yield loss could be estimated as 3%–9% in this study.

Winter oilseed rape yield-loss models for blackleg in the UK have generally estimated losses in relation to disease incidence (percentage of plants infected) on stems prior to harvest [29,30]. In the current study, the relationships between disease severity and the number of pods and seed yield were examined. This study showed considerable variation in the coefficients of the equations (values of the slopes of the regression lines) over the site-years. Previous studies also have shown considerable deviation in yield-loss coefficients between sites. Yield-loss models for blackleg, with losses expressed as the percentage yield loss, have been generated in Australia [25] and in the UK [29,30], but may not be relevant to Alberta because canola types, crop growth characteristics, and disease development can differ greatly between continents. The wide range of climates and agricultural practices under which blackleg occurs [1] likely contribute to the variations in the yield-loss coefficients. The data from the present study were based on production practices that reflected local climates and agricultural practices, and the yield loss coefficients were large. These results highlight the importance of developing appropriate strategies to manage blackleg to minimize yield losses, especially in fields where previous outbreaks of the disease have occurred.

Huang et al. [32] showed that foliar application of flusilazole plus carbendazim decreased stem canker severity more on the oilseed rape “Courage”, with a good yield response, than on the cultivar “Canberra”. Kutcher et al. [18] reported that fungicide treatment (azoxystrobin and vinclozolin) resulted in a yield advantage of 8% in open pollinated blackleg susceptible canola, while there was no yield benefit in a blackleg resistant hybrid canola. In the current study, the application of pyraclostrobin fungicide reduced disease severity and increased yield compared with the control. The reduction in disease severity and yield advantage were greatest when the treatment was applied three times (seedling stage + early flowering stage + late flowering stage). These results suggest that early season fungicide application prevented seedling losses due to blackleg, and later season application mitigated losses in pod numbers, possibly caused by secondary infections. Pyraclostrobin is a quinone outside inhibitor (QoI), blocking electron transport at the quinone-oxidizing site of the cytochrome bc1 complex (complex III) in the mitochondrial respiratory pathway [33,34]. The site-specificity of this chemical increases the probability of the development of fungicide insensitivity in target fungal populations. Indeed, a number of fungal pathogens have shown reduced levels of sensitivity to QoI fungicides due to single amino acid substitutions in the cytochrome b site [33,35,36]. Thus, while a treatment regime consisting of three applications of pyraclostrobin was most effective for reducing blackleg severity in this study, this may not be a cost effective or sustainable approach to managing this disease. Repeated applications of pyraclostrobin would exert selection pressure for insensitivity in *L. maculans* populations, and the rotation of fungicides with different modes of action may be needed to avoid the development of fungicide resistance.

## 4. Materials and Methods

### 4.1. Cultivar Resistance and Blackleg of Canola

The blackleg susceptible canola cultivar “Westar”, the moderately resistant hybrid “1950RR” (Canterra Seeds, Winnipeg, MB, Canada) and the resistant hybrid “46S53RR” (Pioneer, Mississauga, ON), were grown in 2013 at a research field site located at the Crop Diversification Centre North (CDCN), Alberta Agriculture and Forestry, Edmonton, AB (53°39′ N, 113°22′ W). In 2013, the experiment was located at a site that was heavily infested with naturally occurring inoculum of *L. maculans*. When the experiment was repeated in 2014, the experimental plots were located at another site at CDCN where no blackleg had been previously observed; hence, the plots were inoculated by spreading chopped, *L. maculans*-infested canola stubble (taken from a heavily infested field the previous fall) when the plants in the plots were at the cotyledon stage. In addition, the experiment was repeated in 2014 at a site near Namao, AB (53°44′ N, 113°22′ W) where blackleg was prevalent. At each site, the experiment was a randomized complete block (RCB) design with four replications. Each plot consisted of four rows, 6 m in length, spaced 0.25-m apart. Adjacent plots were separated by a 1 m buffer space consisting of bare soil, with 2 m between replications. The seeding dates were 4 June 2013 (CDCN), and 21 May and 11 June 2014 (CDCN and Namao, respectively). The plots were hand-weeded as required, since the use of “Westar” precluded herbicide treatments. To assess the impact of blackleg on canola yield, a 1 m^2^ area of each plot was harvested by sickle and bagged on 5 September 2013 (CDCN), and 12 and 13 September 2014 (CDCN and Namao, respectively), with the remainder of the plots harvested by small-plot combine using a straight cut header on 7 October 2013 and 24 September 2014, and the seed was weighed to determine overall yield. Before harvest, 10 plants were randomly selected, and the number of pods per plant was counted. To assess the relationships of blackleg on pod number and yield, the plants within a 1 m^2^ area were sorted according to their disease severity on a 0–5 scale, where: 0 = no disease in a cross-section of the stem base; 1 = decay on <25% of the cross-sectional area of the crown; 2 = decay on 25%–50% of the cross-section; 3 = decay on 51%–75% of the cross-section; 4 = decay on >75% of the cross section; and 5 = death of the plant [17] (Figure 1). The pod number and seed yield of plants in each disease category were determined and the data were converted to a per plant basis. Average blackleg disease severity per 1 m^2^ plot area also was determined.

In another experiment, the canola hybrid “Invigor 5440” (Bayer CropScience, Calgary, AB, Canada; moderately resistant to blackleg) and “Westar” were seeded on 21 May and 11 June 2015, at two sites located at CDCN and at Namao, respectively. The plots were arranged as a RCB with four replications and inoculated with infested stubble as described above. The plots were harvested (four randomly selected 1 m^2^ areas on 25 and 28 September 2015, respectively, at CDCN and Namao), and sorted as per the blackleg severity categories described above. Pod number and seed yield per plant were recorded to determine their relationship with disease severity. The average disease severity per 1 m^2^ plot area also was determined. The remainder of the plots were harvested by small-plot combine using a straight cut header on 7 October 2013, and 24 September 2014. The number of pods per plant and seed yield were determined as described above.

### 4.2. Foliar Fungicide Application and Blackleg

Headline^®^ fungicide (pyraclostrobin, 250 g/L; BASF, Edmonton, Canada) was sprayed at different frequencies and at different growth stages to create a gradient of disease levels to measure the impact of blackleg on canola and seed yield. Experiments were conducted at three site-years, two at CDCN, Edmonton, in 2014 and 2015, and the other at Namao in 2014. The land preparation and experimental design were as described above. The trials were seeded with the canola hybrid “1950RR”, moderately resistant to blackleg, at 4 g·seed·plot^−1^ (4.44 kg·ha^−1^) with a plot seed drill. In 2014, the plots were seeded on 21 May at CDCN and on 11 June at Namao; in 2015, the plots were seeded on 21 May at CDCN. The plots were inoculated by spreading chopped blackleg-infested canola stubble as described previously. Pyraclostrobin was applied by foliar spray at the seedling stage (2-leaf or less), seedling stage + early (10%) flowering stage, or at the seedling stage + early flowering stage + late flowering (80%) stage at a rate of 92.5 g·a.i.·ha^−1^ in 100 L·ha^−1^ water; a non-sprayed control treatment also was included. Plant counts were conducted on 23 June 2014, and on 27 June 2015, at CDCN, and on 8 July 2014, at Namao. To assess the severity of blackleg, a 1 m^2^ area of each replicate plot was hand-harvested with a sickle and bagged, and disease severity was assessed on a 0–5 scale as described above. Ten plants were randomly selected per replicate plot, and were scored for number of pods per plant. On 17 and 24 September 2014, and 1 October 2015, the remaining plants in the plots at Namao and CDCN, respectively, were harvested with a small plot combine using a straight cut header. The seed was dried and weighed to determine seed yield.

### 4.3. Data Analysis

Data were subjected to analysis of variance following the PROC MIXED of SAS software [37]. The treatment (cultivar or fungicide application regimes as applicable) was considered as fixed effect, and replication and site-year and their interaction as random effects. The response variables were plant count, pod number, and seed yield as applicable. The means were compared following the Tukey-Kramer test, and the mean differences were considered significant at *p* ≥ 0.05. Blackleg severity in all of the experiments was rated on a 0–5 scale, so a non-parametric marginal effects analysis was performed to determine the treatment effects using a SAS macro (available for download at: http://www.ams.med.uni-goettingen.de/download/longit/LD_CI.SAS). The datasets consisted of the following variables: trial, treatment, replication, severity rating, and subject (a unique identifier that is needed for calculating confidence intervals) [38]. Within each dataset, severity was ranked within each replicate and analysis of variance of the ranks was conducted using the PROC MIXED option. The treatment effects on disease severity were represented by the estimated relative effects. Median and mean rank values for each treatment were calculated and treatments with p*i* values outside the confidence interval for a treatment were significantly different from that treatment. For the fungicide trials, the frequency of plants rated 0–5 on the blackleg disease severity scale was calculated and presented in the results.

In addition, for the experiments on cultivar resistance and blackleg, regression analysis was performed to determine the relationships between blackleg severity and the response variables pod number and seed yield, and to estimate the loss of pod number and seed yield for each unit increase in disease severity. In addition, using the y-intercept as an estimate of yield of a variety with no disease, each of the yield data points were transformed into a percentage of the maximum yield, and a regression analysis for yield loss estimation per unit increase of disease severity was performed.

## 5. Conclusions

Blackleg disease severity was lower, and seed yield was higher, in moderately resistant to resistant canola hybrids compared with a susceptible cultivar. The relationships between blackleg and yield or yield components can be explained by linear equations, with seed yield and pod numbers decreasing with increasing disease severity. The selection of resistant cultivars should be recommended to growers, and other disease management strategies such as crop rotation, residue management, and the application of seed and foliar fungicides, merit further study to mitigate yield losses caused by blackleg in canola.

## Figures and Tables

**Table 1:** Median rank and estimated relative effects (p*i*) of the canola hybrids “46S53RR”, “1950RR”, “Invigor 5440”, and the cultivar “Westar” on blackleg severity. Trials were conducted under field conditions near Edmonton (Crop Diversification Centre North; CDCN) and Namao, Alberta in 2013, 2014 and 2015.

Sites/Years	Canola Cultivar/Hybrid	Median Disease Rating	Mean Rank (R*i*)	Estimated Relative Effect (p*i*)	SE	Confidence Interval (95%) for the Estimated Relative Effect	
						Lower	Upper
CDCN and Namao, 2013 and 2014	46S53RR	1.0	25.73	0.35041	0.028	0.30389	0.40557
	1950RR	1.0	33.90	0.46383	0.042	0.39920	0.53134
	Westar	2.0	49.88	0.68576	0.049	0.60437	0.74412
CDCN and Namao, 2015	Invigor 5440	1.0	5.13	0.28906	0.066	0.25906	0.39010
	Westar	2.0	11.88	0.71094	0.058	0.60990	0.74094

The p*i* estimates were determined by SE(R*i*)/N, in which SE(R*i*) is the standard error of the mean rank for the *i*th cultivar as determined in the Mixed procedure of SAS with the lsmeans option. A cultivar/hybrid with a p*i* value outside the confidence interval of any other cultivar/hybrid is significantly different from the other cultivar/hybrid (*p* ≤ 0.05).

**Table 2:** Effect of blackleg on pod number and yield of the canola hybrids “1950RR”, “46S53RR”, “Invigor 5440”, and the cultivar “Westar” under field conditions at five site years near Edmonton (Crop Diversification Centre North; CDCN) and Namao, Alberta, in 2013, 2014 and 2015.

Sites/Years	Canola Cultivar/Hybrid	Resistance	Pods Plant^−1^	Yield t·ha^−1^
^1^ CDCN and Namao, 2013 and 2014	46S53RR	R	285 a	2.66 a
	1950RR	MR	202 b	2.21 a
	Westar	S	137 c	1.21 b
	SE		14.67	0.13
^2^ CDCN and Namao, 2015	Invigor 5440	MR	271 a	1.87 a
	Westar	S	150 b	0.82 b
	SE		7.59	0.08

^1^ Data are the means of four replications × three site-years; ^2^ Data are the means of four replications × two sites. Means in a column followed by the same letter are not significantly different according to the Tukey-Kramer test (*p* ≤ 0.05).

**Table 3:** Effect of pyraclostrobin application (92.5 g·a.i.·ha^−1^) on stand establishment, pod numbers and yield on the canola hybrid “1950RR” at Edmonton (Crop Diversification Centre North; CDCN) in 2014 and 2015, and at Namao, Alberta, in 2014.

Application Timing	Plants Row^−1^	Pods Plant^−1^	Yield t·ha^−1^
**SS**	48 b	153 bc	2.64 a
**SS + EFS**	51 ab	177 ab	2.68 a
**SS + EFS+ LFS**	54 a	207 a	2.69 a
**Untreated control**	37 c	128 c	2.03 b
**SE**	5	33	0.54

Data are the mean of four replications × three site-years. Values in a column followed by the same letter are not significantly different as determined by the Tukey-Kramer test (*p* ≤ 0.05). The treatments consisted of Headline (pyraclostrobin) application at: seedling stage (SS), SS + early flowering stage (EFS), SS + EFS + late flowering stage (LFS), and an untreated control. Active ingredient (a.i.).

**Table 4:** Median rank and estimated relative effects (p*i*) of pyraclostrobin application (92.5 g·a.i.·ha^−1^) on blackleg severity on the canola hybrid “1950RR” at Edmonton (Crop Diversification Centre North; CDCN) in 2014 and 2015, and at Namao, Alberta, in 2014.

Application Timing	Median Disease Rating	Mean Rank (R*i*)	Estimated Relative Effect (p*i*)	SE	Confidence Interval (95%) for the Estimated Relative Effect	
					Lower	Upper
SS	1.5	17.00	0.51563	0.181	0.37590	0.65114
SS + EFS	1.0	15.13	0.45703	0.166	0.32757	0.59778
SS + EFS+ LFS	1.0	10.31	0.30664	0.134	0.21408	0.44832
Untreated control	2.0	23.56	0.72070	0.325	0.57514	0.80638

The p*i* estimates were determined by SE(R*i*)/N, in which SE(R*i*) is the standard error of the mean rank for the *i*th treatment as determined in the Mixed procedure of SAS with the lsmeans option. A treatment with a p*i* value outside the confidence interval of any other treatment is significantly different from the other treatment (*p* ≤ 0.05). The treatments consisted of Headline (pyraclostrobin) application at: seedling stage (SS), SS + early flowering stage (EFS), SS + EFS + late flowering stage (LFS), and an untreated control.
